# Nutri-microbiome epidemiology, an emerging field to disentangle the interplay between nutrition and microbiome for human health

**DOI:** 10.1093/procel/pwad023

**Published:** 2023-04-26

**Authors:** Wanglong Gou, Zelei Miao, Kui Deng, Ju-Sheng Zheng

**Affiliations:** Westlake Intelligent Biomarker Discovery Lab, Westlake Laboratory of Life Sciences and Biomedicine, Hangzhou 310024, China; Research Center for Industries of the Future, Key Laboratory of Growth Regulation and Translational Research of Zhejiang Province, School of Life Sciences, Westlake University, Hangzhou 310030, China; Institute of Basic Medical Sciences, Westlake Institute for Advanced Study, Hangzhou 310024, China; Westlake Intelligent Biomarker Discovery Lab, Westlake Laboratory of Life Sciences and Biomedicine, Hangzhou 310024, China; Research Center for Industries of the Future, Key Laboratory of Growth Regulation and Translational Research of Zhejiang Province, School of Life Sciences, Westlake University, Hangzhou 310030, China; Institute of Basic Medical Sciences, Westlake Institute for Advanced Study, Hangzhou 310024, China; Westlake Intelligent Biomarker Discovery Lab, Westlake Laboratory of Life Sciences and Biomedicine, Hangzhou 310024, China; Research Center for Industries of the Future, Key Laboratory of Growth Regulation and Translational Research of Zhejiang Province, School of Life Sciences, Westlake University, Hangzhou 310030, China; Institute of Basic Medical Sciences, Westlake Institute for Advanced Study, Hangzhou 310024, China; Westlake Intelligent Biomarker Discovery Lab, Westlake Laboratory of Life Sciences and Biomedicine, Hangzhou 310024, China; Research Center for Industries of the Future, Key Laboratory of Growth Regulation and Translational Research of Zhejiang Province, School of Life Sciences, Westlake University, Hangzhou 310030, China; Institute of Basic Medical Sciences, Westlake Institute for Advanced Study, Hangzhou 310024, China

**Keywords:** microbiome, nutrition, human health, epidemiology

## Abstract

Diet and nutrition have a substantial impact on the human microbiome, and interact with the microbiome, especially gut microbiome, to modulate various diseases and health status. Microbiome research has also guided the nutrition field to a more integrative direction, becoming an essential component of the rising area of precision nutrition. In this review, we provide a broad insight into the interplay among diet, nutrition, microbiome, and microbial metabolites for their roles in the human health. Among the microbiome epidemiological studies regarding the associations of diet and nutrition with microbiome and its derived metabolites, we summarize those most reliable findings and highlight evidence for the relationships between diet and disease-associated microbiome and its functional readout. Then, the latest advances of the microbiome-based precision nutrition research and multidisciplinary integration are described. Finally, we discuss several outstanding challenges and opportunities in the field of nutri-microbiome epidemiology.

## Introduction

Human microbiota inhabit across various anatomical body sites such as the skin, oral mucosa, saliva, gastrointestinal tract, respiratory tract, urogenital tract, and the mammary gland (Sender et al., 2016), with the gastrointestinal tract being the most heavily studied body site for human microbiome research. Trillions of microbiota inhabit in the human gastrointestinal tract, which are continuously perturbed by daily dietary intake (Sender et al., 2016; Johnson et al., 2019). The complex interaction between diet, nutrition, and the gut microbiota plays an essential role in modulating the host health (Valdes et al., 2018a; Kolodziejczyk et al., 2019). For example, gut microbiota could metabolize dietary nutrients into functional metabolites, such as disease-causing metabolite trimethylamine N-oxide (TMAO), which was associated with higher risk of the cardiovascular diseases (Senthong et al., 2016; Li et al., 2017; Yu et al., 2019; Lee et al., 2021; Wei et al., 2022), or disease protective metabolite short-chain fatty acids (SCFA) that stimulate the secretion of glucagon-like peptide-1 and regulate glucose metabolism (He et al., 2020).

The advent of high-throughput DNA sequencing technologies (such as 16S, ITS, and metagenomics sequencing) enables profiling of the microbial communities, which facilitates the integration of microbiome big-data with large-scale epidemiological studies or consortium, such as the American Gut Project (McDonald et al., 2018), Dutch LifeLines-DEEP study (Tigchelaar et al., 2015), FINRISK study (Borodulin et al., 2018), and Westlake Gut Project (Gou et al., 2022). Moreover, it is widely recognized about the profound variation in the host response to the identical diet or meal (Zeevi et al., 2015; Berry et al., 2020; Ma et al., 2021), which stimulates numerous research to explore the potential mechanism, with gut microbiome becoming the spotlight of research in the field recently (Zeevi et al., 2015; Korem et al., 2017; Johnson et al., 2019; Mendes-Soares et al., 2019; Berry et al., 2020; Asnicar et al., 2021; Suez et al., 2022). Therefore, microbiome (especially gut microbiome)-based precision nutrition research is becoming one of the frontiers in the field of nutri-microbiome epidemiology.

In this review, we will first summarize the findings of microbiome epidemiological studies that investigate the associations of microbiome and microbial metabolites with diet, nutrition, and human diseases. Then, we will review the current progress of the microbiome-based precision nutrition research. Finally, current limitations, challenges, and perspectives of the nutri-microbiome epidemiology field are presented.

## Role of diet and nutrition in shaping the gut microbiome

**Figure 1.**
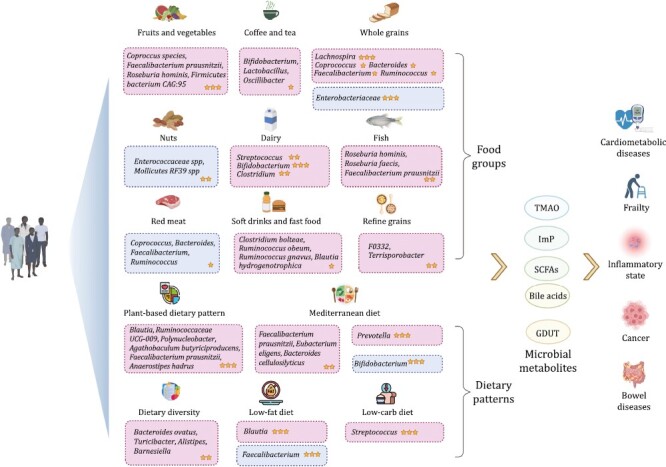
**Associations of microbiome and microbial metabolites with diet, and human diseases.** Nutri-microbiome epidemiology studies identify the associations of microbiome and microbial metabolites with diet, and human diseases. Box colors indicate the direction of association of microbiome with diet (pink, positive; blue, negative). The number of stars indicates the level of evidence for the results. Specifically, one star indicates that the results were from cross-sectional studies. Two stars indicates that the results were from prospective cohort studies, and three stars indicates that the results were from clinical trials or could be replicated in different studies. This figure was created with BioRender.com.

Host diet is key for the symbiotic role of the gut microbiota. On one hand, the gut microbiota depends on the host intake of nutrients for their own survival, on the other, many gut microbes directly participate in the digestive process, producing a variety of nutrients involved in the host metabolism and biology process (Valdes et al., 2018b; Kolodziejczyk et al., 2019). Data from human cohorts and clinical trials are accumulating recently, showing how specific food types (including food groups and nutrients) and dietary patterns, influence the gut microbiome and subsequently host health (Fig. 1 and Table 1).

**Table 1.** Literature about diet-gut microbiome associations and their roles in the host health^*^.

Study design	Dietary feature	Study	Sample size	Duration of follow-up	Country	Sequencing	Health outcome
Cross-sectional study	Vegan diet	Wu et al. (2016)	31	N/A	USA	16S rRNA	N/A
			Plasma metabolome of vegans differed markedly from omnivores but the gut microbiota was surprisingly similar. Higher consumption of fermentable substrate in vegans was not associated with higher levels of fecal SCFAs.				
	Habitual diet (foods, food groups, nutrients, and dietary patterns)	Zhernakova et al. (2016)	1,135	N/A	Netherlands	Metagenomics	N/A
			Sixty dietary factors were associated with the gut microbiome, including energy (kcal), intake of carbohydrates, proteins and fats, and of specific food items such as bread and soft drinks.				
	N-3 fatty acids	Menni et al. (2017)	876	N/A	UK	16S rRNA	N/A
			Total omega-3 fatty acids and docosahexaenoic acid (DHA) were correlated with high microbial diversity. DHA was positively associated with operational taxonomic units from the *Lachnospiraceae* family.				
	Vegetarian diet	Losasso et al. (2018)	101	N/A	Italy	16S rRNA	N/A
			Vegetarians had a significantly greater microbial richness and a higher abundance of *Bacteroidetes* related operational taxonomic units compared to omnivorous.				
	Dietary patterns identified using unsupervised hierarchical clustering and food groups	Bolte et al. (2021)	1,425	N/A	Netherlands	Metagenomics	Inflammation
			Diet-gut microbiome associations are consistent across patients with intestinal disease and the general population. Higher intake of animal foods, processed foods, alcohol, and sugar, is associated with higher levels of intestinal inflammatory markers. The opposite was found for plant foods and fish.				
	Habitual diet (foods, food groups, nutrients, and dietary patterns)	Asnicar et al. (2021)	1,098	N/A	UK and USA	Metagenomics	Cardiometabolic blood markers
			Habitual diet is linked to overall and feature-level composition of the gut microbiome. The panel of microbial species associated with healthy habitual diet overlapped with those associated with favorable cardiometabolic and postprandial markers.				
	Habitual diet (foods, food groups, nutrients, and dietary patterns)	Breuninger et al. (2021)	1,992	N/A	German	16S rRNA	Metabolic diseases or risk factors
			A panel of microbial species, including *Faecalibacterium*, *Lachnospiracea incertae sedis*, *Gemmiger*, and *Roseburia*, was associated with higher Alternate Healthy Eating Index and MedDiet Score and a higher intake of food items such as fruits, vegetables, legumes, and whole grains, and a lower prevalence of T2D.				
	Ultra-processed food	Cuevas-Sierra A et al. (2021)	359	N/A	Spain	16S rRNA	N/A
			A consumption higher than five servings per day of ultra-processed food may affect gut microbiota composition differently in women and men				
	Habitual diet (food groups)	Wang et al. (2022a)	2,772	N/A	China	16S rRNA	T2D
			Microbial genera that were favorable for the glycemic trait were consistently associated with healthy dietary habits (higher consumption of vegetable, fruit, fish, and nuts).				
	Dietary diversity	Huang et al. (2022)	128	N/A	China	16S rRNA	Cardiometabolic disease biomarkers
			Dietary variety was correlated with higher gut microbial diversity. The combination of *Alistipes*, *Roseburia*, and *Barnesiella* could moderately predict dietary variety level.				
Prospective study	N-6 PUFAs	Miao et al. (2020)	1,591	A median follow-up of 6.2 years	China	16S rRNA	T2D
			Gut microbial diversity acted as a potential mediator in the association between γ-linolenic acid and T2D risk. Seven genera were enriched in quartile 1 of gamma-linolenic acid and in participants without T2D.				
	Fruit and vegetable	Jiang et al. (2020)	8,505	A median follow-up of 6.2 years	China	16S rRNA	T2D
			Fruit intake, but not vegetable, was associated with gut microbiota diversity and composition. The fruit-microbiota index (created from 31 fruit-related microbial features) was positively associated with fruit intake and inversely associated with T2D risk.				
	Habitual diet (food groups)	Gou et al. (2021)	1,832	A median follow-up of 6.2 years	China	16S rRNA	T2D
			Tea drinking was inversely associated with a microbiome risk score calculated based on 14 microbial features associated with T2D.				
	Dairy	Shuai et al. (2021)	1,780	A median follow-up of 6.2 years	China	16S rRNA	Cardiometabolic health biomarkers
			Dairy consumption is associated with the gut microbial composition and a higher alpha-diversity, which were inversely associated with blood triglycerides, while positively associated with high-density lipoprotein cholesterol.				
	Healthy diet score and food groups	Yu et al. (2021)	1,920	5.2–20.5 years of follow-up	China	16S rRNA	N/A
			Among healthy Chinese adults, long-term diet quality is positively associated with fecal microbiome diversity and abundance of fiber-fermenting bacteria.				
	MedDiet and food groups	Wang et al. (2021)	307	2 pairs of fecal samples collected 6 months apart	USA	Metagenomics	Cardiometabolic disease risk
			A healthy Mediterranean-style dietary pattern is associated with specific functional and taxonomic components of the gut microbiome, and that its protective associations with cardiometabolic health vary depending on microbial composition (e.g., the relative abundance of *Prevotella copri*).				
	Dietary diversity	Xiao et al. (2022)	3,236	Over 3 years of follow-up	China	16S rRNA	Glycemic and inflammatory phenotypes
			High dietary diversity is associated with the gut microbial diversity and composition. Both the dietary diversity and diversity-related microbial features were correlated with host circulating secondary bile acids.				
	Plant-based dietary pattern and food groups	Miao et al. (2022)	3,096	3 years	China	16S rRNA	Cardiometabolic biomarkers
			Long-term and short-term plant-based dietary pattern were differently associated with gut microbial diversity and composition. Microbes related to long-term plant-based dietary pattern showed association with future cardiometabolic biomarkers.				
Intervention study	Red wine	Queipo-Ortuño et al. (2012)	10 healthy volunteers	4 weeks	Spain	PCR	Cardiometabolic blood markers
			The daily consumption of red wine polyphenol for 4 weeks significantly increased the abundance of *Bifidobacterium*. Changes in cholesterol and C-reactive protein concentrations were linked to changes in the bifidobacteria number.				
		Moreno-Indias et al. (2016)	10 obese individuals	30 days	Spain	PCR	Metabolic syndrome markers
			In the metabolic syndrome patients, red wine polyphenols significantly increased the number of butyrate-producing bacteria. The changes in gut microbiota in these patients contributed to the improvement in the metabolic syndrome markers.				
	Whole grains	Vanegas et al. (2017)	81 healthy adults	6 weeks	USA	16S rRNA	Inflammatory makers
			Substituting whole grains for refined grains for 6 weeks increased *Lachnospira* and decreased proinflammatory *Enterobacteriaceae*, and had modest positive effects on acute innate immune response.				
	Omega-3 fatty acids	Watson et al. (2017)	22 healthy volunteers	8 weeks	UK	16S rRNA	N/A
			There were no significant changes in alpha- or beta-diversity, or phyla composition, associated with omega-3 fatty acid supplementation. However, a reversible increased abundance of several genera, including *Bifidobacterium*, *Roseburia*, and *Lactobacillus* was observed with omega-3 fatty acid intervention.				
	Dietary fat	Wan et al. (2019)	217 young adults	6 months	China	16S rRNA	Blood lipids and Inflammatory factors
			The low-fat diet was associated with increased microbial alpha-diversity and abundance of *Blautia* and *Faecalibacterium*. Change in relative abundance of *Blautia* was negatively associated with the changes in serum total cholesterol, low-density lipoprotein cholesterol, and non-high-density lipoprotein cholesterol.				
	MedDiet	Ghosh et al. (2020)	612 non-frail or pre-frail participants	6 months	Italy, UK, Netherlands, Poland, and France	16S rRNA	Frailty
			Adherence to the MedDiet led to increased abundance of specific taxa that were positively associated with several markers of lower frailty and improved cognitive function.				
		Rinott et al. (2022)	286 participants with abdominal obesity/dyslipidemia were	1 year	Israel	16S rRNA and metagenomics	Cardiometabolic disease biomarkers
			The level of adherence to the Green-MedDiet was associated with the changes in microbiome composition, and changes in gut microbial features mediated the association between adherence to the Green-MedDiet and body weight and cardiometabolic risk reduction.				
	Non-nutritive sweeteners	Suez et al. (2022)	120 healthy participants	14 days	Israel	Metagenomics	Glucose tolerance
			Administered saccharin, sucralose, aspartame, and stevia sachets for 2 weeks in doses lower than the acceptable daily intake distinctly altered stool and oral microbiome, whereas saccharin and sucralose significantly impaired glycemic responses.				

^*^Except for a few pioneer and intervention studies, most of the studies listed in the table were published within past 5 years, and with sample size larger than 1,000.

### Food types

Consumptions of vegetables and fruits were associated with higher abundance of SCFA producers. For example, fruit and vegetable intakes were both positively associated with *Coprococcus* species, *Faecalibacterium prausnitzii*, *Roseburia hominis,* and *Firmicutes bacterium CAG:95* across several studies (Liu et al., 2019; Jiang et al., 2020; Asnicar et al., 2021; Breuninger et al., 2021). Higher proportions of SCFA-producing bacteria have beneficial effects on the human host, including the regulation of energy balance, immune system, and glucose metabolism (Deleu et al., 2021). Gut microbe that was favorable for the glycemic traits or T2D was associated with higher consumption of vegetables and fruits (Jiang et al., 2020; Wang et al., 2022a). Moreover, gut microbiota has been shown to mediate the effect of vegetables on the white blood cell profiles (Menni et al., 2021).

The beneficial role of vegetables and fruits may be owing to the contribution of fiber and flavonoids in diet-microbe cross-talk. Fiber is a non-digestible carbohydrate found in plants and provides the natural sources for fecal SCFAs. Therefore, high-fiber diet was commonly investigated in the context of disease treatment, such as inflammatory bowel disease (Wedlake et al., 2014; Deleu et al., 2021), cancer (Lam et al., 2021), and Type 2 diabetes (T2D) (Ren et al., 2018). Some randomized controlled trials involving fiber supplementation successfully altered microbial activity (i.e., SCFA production) and provided symptomatic relief or disease remission (Ren et al., 2018; Lam et al., 2021), however, some other trials failed (Wedlake et al., 2014; Deleu et al., 2021). Anthocyanins, a particular class of flavonoids, are enriched in fruits and vegetables, and could be metabolized by the intestinal microbiota (Faria et al., 2014; Boronat et al., 2021). Moreover, anthocyanins and their metabolites could enhance the growth of beneficial bacteria (e.g., *Bifidobaterium* and *Lactobacillus*) and reduce a group of potentially harmful bacteria, such as *Clostridium histolyticum* (Sánchez-Patán et al., 2012; Faria et al., 2014). Similar results in the *Bifidobaterium* have also been described in human volunteers after 6-week consumption of a blueberry drink (Vendrame et al., 2011). In addition, a recent study examined the microbial associations of circulating equol, a metabolite of dietary soy isoflavones, and found higher *Alistipes senegalensis* and *Coprococcus catus* but lower abundances of *Ruminococcus gnavus* were associated with equols (Wu et al., 2022b).

Polyphenol-rich foods, such as coffee and tea, could also affect the gut microbiome and influence host health. For example, coffee and tea were positively associated with *Bifidobacterium*, *Lactobacillus*, and *Oscillibacter* (Singh et al., 2017), and were inversely associated with proinflammatory pathways (Bolte et al., 2021). Tea consumption was inversely associated with unfavorable gut microbial profiles for T2D (Gou et al., 2021). Red wine polyphenols have been shown to modulate the gut microbiota (e.g., could increase *F. prausnitzii* and *Roseburia*; decrease *E*. *coli* and *Bifidobacterium*) and improve the metabolic profiles in healthy and obese participants (Queipo-Ortuño et al., 2012; Moreno-Indias et al., 2016; Bolte et al., 2021).

Refined grains showed different gut microbial associations from above healthy plant-based foods, and were associated with higher abundance of opportunistic bacterial genera such as *F0332* and *Terrisporobacter* (Miao et al., 2022). In a 6-week randomized controlled trial, substituting whole grains for refined grains decreased levels of proinflammatory *Enterobacteriaceae* and increased SCFA-producing bacteria *Lachnospira* (Vanegas et al., 2017). It might be due to fewer fiber contained in the refined grains as they usually have seed coat removed. Previous studies also demonstrated that barely whole grains were more fermentable that refined wheat, and thus had a more effective SCFA production pattern (Nordlund et al., 2012; Vetrani et al., 2016).

Nuts and seeds also had a strong association with composition of gut microbiome. *Roseburia hominis* was positively associated with nuts intake, and showed beneficial effects on glucose metabolism by increasing butyrate production (Bolte et al., 2021). *Enterococcaceae* spp and *Mollicutes* RF39 spp had inverse associations with nuts intake and were potentially correlated to a unfavorable glycemic profile (Wang et al., 2022a; Miao et al., 2022). n-6 polyunsaturated fatty acids (PUFAs) is mainly enriched in nuts, a prospective cohort study shown that n-6 PUFAs was associated with lower microbial diversity and a higher risk of T2D, accordingly (Miao et al., 2020). It indicated that the beneficial association between nuts intake and glucose metabolism might be mediated by gut microbiota.

Consumption of total dairy and fermented dairy (e.g., yogurt) showed strong associations with high microbial alpha-diversity, lactic bacteria (*Leuconostoc mesenteroides* and *Lactococcus lactis*), and fermentation to butanediol pathway (Zhernakova et al., 2016; Bolte et al., 2021; Shuai et al., 2021; Yu et al., 2021). The consumption of milk was reported to be positively associated with the abundance of *Streptococcus*, *Bifidobacterium*, and *Clostridium* (Shuai et al., 2021). Of note, the dairy-*Bifidobacterium* association was consistently reported in several studies (Y. Zhernakova et al., 2016; Liu et al., 2019; Shuai et al., 2021). Dairy-favorable gut microbial features were inversely associated with blood triglycerides, while positively associated with high-density lipoprotein cholesterol (Shuai et al., 2021).

Several microbial associations have been identified for fish, including positive associations with *R. hominis*, *Roseburia faecis,* and *F. prausnitzii* (Bolte et al., 2021; Yu et al., 2021). Available evidence also highlights the potential importance of the dietary n-3 PUFAs, enriched in fish, to modulate the gut microbial composition. For example, after n-3 PUFA supplementation, a decrease in *Faecalibacterium,* and an increase in the *Bacteroidetes* and the production of SCFA have been observed (Costantini et al., 2017). Another cohort of 876 middle-aged and elderly women showed that n-3 PUFAs were correlated with microbial features belonging to *Lachnospiraceae* family, *Ruminococcaceae* family, and *Bacteroidetes* phylum (Menni et al., 2017). Nevertheless, a randomized cross-over trial including 22 middle-aged, healthy volunteers found that 8-week n-3 PUFA supplementation did not significantly change the gut microbial alpha-diversity, overall structure, or phyla abundance (Watson et al., 2017). On the other hand, n-3 PUFAs may interact with host genetics to affect gut microbiome and further link to diseases. A recent study found that higher n-3 PUFAs were prospectively associated with gut microbial features (diversity and genera) only among rs1527483-GG carriers, n-3 PUFAs-related gut microbial features were associated with blood lipids (Miao et al., 2022).

Red meat showed opposite taxonomic associations with above healthy plant-based foods. For example, *Coprococcus*, *Bacteroides*, *Faecalibacterium*, and *Ruminococcus* were inversely associated with processed red meat, and positively associated with fruit, whole grains, and fiber intake (Breuninger et al., 2021). Diet high in red meat may lead to the production of harmful bacterial metabolites such as TMAO leading to a disruption of the gut microbiota and increased risk of various diseases such as cardiovascular diseases and colon cancer (Koeth et al., 2013; Zaramela et al., 2019; Li et al., 2022).

Of note, other processed foods such as soft drinks and fast food had shown similar microbial associations with processed meat and were consistently linked to a higher abundance of *Clostridium bolteae*, *Ruminococcus obeum*, *R. gnavus*, and *Blautia hydrogenotrophica* (Breuninger et al., 2021). It indicated an impact of processed/ultra-processed foods on the human gut microbiome. Recent observational studies found that ultra-processed food intake was positively associated with *Acidaminococcus*, *Butyrivibrio*, *Gemmiger*, *Shigella*, *Anaerofilum*, *Parabacteroides*, *Bifidobacterium* in women, and with *Granulicatella* and *Blautia* in men (Cuevas-Sierra et al., 2021). Food additives, such as emulsifiers, which are commonly used in the processed foods, had a potential negative impact on the gut microbiota using mice and *in vivo* models (Chassaing et al., 2015; Naimi et al., 2021). Another kind of commonly used food additive artificial sweeteners may impact gut microbiota and human health as well. A randomized controlled trial performed among 120 healthy participants showed that oral supplementation with the sweeteners sucralose, saccharin, stevia, and aspartame induced perturbations of glucose tolerance, which might be mediated by compositional and functional changes in the gut microbiota (Suez et al., 2022). Currently, this field is severely under-researched in human epidemiology studies and further researches are needed to address the underlying mechanism of how food processing could influence gut microbiota and human health.

Collectively, these findings demonstrate that specific food types are closely correlated with gut microbial profiles and human health. Shared and distinct microbial associations have been reported for different food types.

### Dietary patterns

Plant-based dietary pattern is growing in popularity due to its beneficial effects for human health, including reduced risk of T2D (Qian et al., 2019), cancer (Molina-Montes et al., 2020), and cardiovascular diseases (Kim et al., 2019). Early studies examined the impact of plant-based dietary pattern on the gut microbiome by characterizing the gut microbial composition of vegans or vegetarians compared with omnivores (De Filippis et al., 2016; Wu et al., 2016; Losasso et al., 2018). The vegetarian diets including vegan diets were reported to be associated with higher richness of gut microbiome and were associated with increased abundance of *Bacteroidetes* and decreased abundance of *Firmicutes* (Losasso et al., 2018). Vegetarian and vegan diets were also associated with higher abundance of *Lachnospira* and *Prevotella* in the gut, whereas omnivorous diets were associated with much lower levels (De Filippis et al., 2016). However, another US study found negligible differences in gut microbial community composition between vegans and omnivores in an urban environment (Wu et al., 2016).

Recent large-scale studies based on free-living individuals found that plant-based dietary pattern, as assessed using multiple 24-hour food recalls or food frequency questionnaires, were consistently associated with gut microbial alpha-diversity and overall composition (Asnicar et al., 2021; Bolte et al., 2021; Miao et al., 2022). These findings motivated the detailed exploration of plant-based diet-related microbial taxa and functional pathways. For example, habitual plant-based dietary pattern was associated with several commensals capable of SCFA production, including genus (*Blautia*, *Ruminococcaceae* UCG-009, and *Polynucleobacter*) and species (*Agathobaculum butyriciproducens*, *F. prausnitzii,* and *Anaerostipes hadrus*) (Asnicar et al., 2021; Miao et al., 2022). Consistently, Bolte et al. found the associations between plant-based foods and several carbohydrate fermentation pathways (Bolte et al., 2021).

Mediterranean diet (MedDiet) is characterized by high intakes of vegetables, fruits, nuts, and fish, and was associated with gut microbiome (De Filippis et al., 2016; Mitsou et al., 2017; Garcia-Mantrana et al., 2018; Merra et al., 2021; Wang et al., 2021a; Rinott et al., 2022). Using a longitudinal microbiome data, Wang et al. identified several gut microbial functional and taxonomic signatures for the MedDiet adherence (Wang et al., 2021a). At the taxonomic level, they found that *F. prausnitzii*, *Eubacterium eligens*, and *Bacteroides cellulosilyticus* were positively associated with MedDiet adherence. At the functional level, MedDiet adherence was associated with plant polysaccharide degradation, SCFA production, and pectin metabolism (Wang et al., 2021a). Moreover, in a 12-month randomized controlled trial including 612 participants, MedDiet could induce the enrichments of genus *Prevotella* and enzymatic functions involved in branched-chain amino acid degradation, and reduce the abundance of genus *Bifidobacterium* and enzymatic functions responsible for branched-chain amino acid biosynthesis (Ghosh et al., 2020). Those microbial features were also thought to mediate the effects of MedDiet on cardiometabolic health (Shankar Ghosh et al., 2020; Rinott et al., 2022).

A low-carb high-fat ketogenic diet is a diet that restricts carbohydrates intakes, primarily derived from sugary foods and refined grains, which could alter human gut microbiota. A short-term intervention with an isocaloric low-carb diet with increased protein content shifted the gut microbiota in obese participants with nonalcoholic fatty liver disease, including an increase in folate-producing *Streptococcus* and decreased fecal concentrations of SCFAs (Mardinoglu et al., 2018). Low-carb diet-associated gut microbiota could inhibit bifidobacterial growth, reduce the levels of intestinal inflammation and thus benefit metabolic health (Ang et al., 2020). Similar as the low-carb diet, low-fat diet was also demonstrated to be highly associated with gut microbiome. For example, a 6-month randomized controlled-feeding trial including 217 healthy young adults found that low-fat diet could increase gut microbial alpha-diversity, *Blautia* and *Faecalibacterium*, and reduce the blood proinflammatory markers (Wan et al., 2019).

Recently, dietary diversity has been linked to gut microbial profiles in the human cohort studies (Huang et al., 2022; Xiao et al., 2022). A diverse diet was consistently associated with a high microbial alpha-diversity, and a high abundance of *Bacteroides ovatus*, *Turicibacter*, *Alistipes*, and *Barnesiella* (Huang et al., 2022; Xiao et al., 2022). Nevertheless, data about the dietary diversity and gut microbiome is still sparse and more research in this field is warranted to further demonstrate the interplay between diversity of diet and gut microbiome profiles for host health.

## Diet, nutrition, and microbiome across body sites beyond gut

Diet and nutrients could also affect the microbiota from other body sites, such as oral microbiota, vaginal microbiota, and human milk microbiota (Table 2). Oral microbiota also attracts attention given their close connection with diet and health (Lamont et al., 2018). Oral microbial alpha-diversity was positively associated with tea intake and inversely associated with total carbohydrates, glycemic load (GL), starch, lactose, and sucrose intake in US elderly populations (Peters et al., 2018; Millen et al., 2022). One study among Thailand children showed that frequently consumed snacks were associated with higher alpha-diversity of oral gut microbiota, which could predict early childhood caries (Wu et al., 2022a). Oral microbial beta-diversity was associated with tea intake, sweet treat consumption, total carbohydrates, fiber, GL, sucrose, and galactose (Peters et al., 2018; Lommi et al., 2022; Millen et al., 2022). Diet and nutrients were also associated with specific oral bacterial patterns (Esberg et al., 2021; Shaalan et al., 2022). For example, one study found that sucrose intake was associated with one oral bacterial pattern defined by the highest predicted sugar-related metabolic pathways and lowest species diversity, and caries status in Swedish adults (Esberg et al., 2021). Another study showed that consumptions of sugary snacks combined with reduced consumption of fish/shellfish and nuts were associated with one subtype of oral microbiome that was more represented in participants with T2D (Shaalan et al., 2022). For specific oral microbes, Peters et al. found that higher tea intake was associated with higher abundance of *Fusobacteriales*, *Clostridiales*, and *Shuttleworthia satelles*, and lower abundance of *Bifidobacteriaceae*, *Bergeyella*, *Lactobacillales*, and *Kingella oralis*. They also reported that higher coffee intake was associated with higher abundance of *Granulicatella* and *Synergistetes*, although it was not associated with alpha- or beta-diversity among the US elderly population (Peters et al., 2018). Higher sweet treat consumption was associated with higher abundance of *Streptococcus*, *Prevotella*, *Veillonella*, and *Selenomonas*, and associated with activated nitrate reduction IV and gondoate biosynthesis pathways among Finland children (Lommi et al., 2022). Thus, current evidence supports the associations of the intakes of tea, coffee, sugary foods, and carbohydrates with oral microbiota.

**Table 2.** Literature for diet, nutrition, and microbiome across body sites other than gut*.

Study design	Body site	Study	Sample size	Country	Sequencing	Duration of follow-up	Diet and nutrition	Health outcome	Method
**Cross-sectional study**	Oral cavity	Peters et al. (2018)	938	USA	16S rRNA	N/A	Coffee and Tea Intake	N/A	Negative binomial generalized linear models
			Higher tea intake was associated with greater oral microbiota richness and diversity; higher tea intake was associated with higher abundance of *Fusobacteriales*, *Clostridiales*, and *Shuttleworthia satelles*, and lower abundance of *Bifidobacteriaceae*, *Bergeyella*, *Lactobacillales*, and *Kingella oralis*; higher coffee intake was associated with higher abundance of *Granulicatella* and *Synergistetes*.						
		Esberg et al. (2021)	427	Sweden	16S rRNA	N/A	Sucrose consumption	Caries	Partial least square regression discrimination analysis (PLS-DA), PCA
			Sucrose intake was associated with one oral bacterial pattern defined by highest predicted sugar-related metabolic pathways and lowest species diversity, and caries status in Swedish adults.						
		Millen et al. (2022)	1,204	USA	16S rRNA	N/A	Total carbohydrates, starch, monosaccharides, disaccharides, fiber, or GL	N/A	PERMANOVA, linear regression
			Total carbohydrates, GL, starch, lactose, and sucrose intakes were associated with alpha-diversity. Total carbohydrates, fiber, GL, sucrose, and galactose were associated with beta-diversity.						
		Wu et al. (2022a)	177	Thailand	16S rRNA	N/A	Snack consumption	Early childhood caries	LASSO penalized logistic regression model
			Frequently consumed snacks were associated with higher alpha-diversity of oral gut microbiota, which could predict early childhood caries among Thailand children.						
		Lommi et al. (2022)	700	Finland	16S rRNA	N/A	Sweet treats	N/A	ANOVA, ANCOVA, PERMANOVA
			Sweet treat consumption was associated with beta-diversity. Higher sweet treat consumption was associated with higher abundance of *Streptococcus*, *Prevotella*, *Veillonella*, and *Selenomonas*, and were associated with activated nitrate reduction IV and gondoate biosynthesis pathways among Finland children.						
	Milk	LeMay-Nedjelski et al. (2021)	93	Canada	16S rRNA	N/A	Intake of polyunsaturated fat, fiber from grains	N/A	Multivariable Poisson regression model
			Maternal consumptions of fiber and fat, and mother’s infant feeding practice (frequency of direct breastfeeding), were also significantly associated with human milk microbiota.						
	Vagina	Dall’Asta et al. (2021)	24	Italy	16S rRNA	N/A	Pre-pregnancy intake of animal-sourced protein, total carbohydrates and sugars	N/A	Spearman correlation
			Higher pre-pregnancy intake of animal-sourced protein was inversely (harmfully) associated with a lactobacilli-dominated vaginal microbiota, while intakes of total carbohydrates and sugars were beneficial for healthy vaginal microbiota.						
		Rosen et al. (2022)	634	USA	16S rRNA	N/A	Dairy, fruit, vitamin D, fiber and, yogurt	N/A	Adjusted Poisson models with robust variance estimators
			The assumption of low-fat dairy was associated with a beneficial vagitype with predominance of *Lactobacillus* species.						
**Prospective study**	Oral cavity	Shaalan et al. (2022)	121	Spain	16S rRNA	N/A	Elements of the MedDiet	T2D, body mass index	PERMANOVA test, DESeq2 negative binomial tests, Dirichlet multinomial mixtures
			Consumption of sugary snacks combined with reduced consumption of fish/shellfish and nuts was associated with one subtype of oral gut microbiota pattern that was more represented in participants with T2D.						
	Milk	Liu et al. (2022)	53	China	16S rRNA	First 6 months postpartum	Maternal diet such as tuber, nutrients such as carbohydrates and vitamin B12	N/A	Spearman correlations
			Tuber intake was positively associated with the abundance of *Neisseria* and *Cutibacterium* in the breast milk. Carbohydrate was inversely associated with the abundance of *Aquabacterium*, and vitamin B12 was positively correlated with *Coprococcus*.						

*The studies listed in the table were published within past 5 years.

Human breast milk benefits infants’ immune system maturation and gastrointestinal development by providing nutrients, probiotics, and other bioactive molecules. Liu et al. showed that maternal diets were associated with the breast milk microbial composition in a Chinese population (Liu et al., 2022). For example, tuber was positively associated with the abundance of *Neisseria* and *Cutibacterium* in the breast milk; carbohydrate intake was inversely associated with the abundance of *Aquabacterium*; and vitamin B12 was positively associated with *Coprococcus* (Liu et al., 2022). Maternal consumption of fiber and fat, and mother’s infant feeding practice (frequency of direct breastfeeding) were associated with human milk microbiota (LeMay-Nedjelski et al., 2021). These studies suggest that the breast milk microbiota are influenced by maternal intake of tuber, fiber, and fat, as well as mother’s infant feeding practice.

The balance of vaginal microbiota was crucial for maintaining maternal-fetal health (France et al., 2022). Through investigating the effects of pre-pregnancy diet on vaginal microbiota composition, Dall’Asta et al. found that higher pre-pregnancy intakes of animal-sourced protein were inversely (harmfully) associated with a lactobacilli-dominated vaginal microbiota, while intakes of total carbohydrates and sugars were beneficial for healthy vaginal microbiota (Dall’Asta et al., 2021). Rosen et al. explored the associations between prenatal diet and vaginal microbiota composition and showed that the assumption of low-fat dairy was associated with a beneficial vagitype with predominance of *Lactobacillus* species (Rosen et al., 2022). These studies demonstrate the importance of pre-pregnancy (total carbohydrates and sugars) and prenatal diet (low-fat dairy) for healthy vaginal microbiota.

## Microbial metabolites provide functional connection between diet, microbiome, and human health

Microbial metabolites act as important intermediates in the crosstalk between gut microbiota and host health (Fan and Pedersen, 2021; Krautkramer et al., 2021; Wang et al., 2022c). Microbial metabolites play essential roles in host metabolic homeostasis, immune functions, and neuromodulation (Fan and Pedersen, 2021; Krautkramer et al., 2021). Through producing a diverse array of metabolites, gut microbiota could even exert its functions in distant organs of human body, such as liver, lung, and brain (Van de Wouw et al., 2017; Tripathi et al., 2018; Dang and Marsland, 2019; Fan and Pedersen, 2021). The production of microbial metabolites depends, at least partially, on the availability of dietary substrate, in which bacteria ferment dietary macronutrients and micronutrients into functional metabolites. In this section, we summarize current epidemiological studies linking the associations between diet, microbial metabolites, and human health (Table 3).

**Table 3.** Literature about diet, microbial metabolites, and human health.

Study design	Metabolites	Study	Sample size	Country	Sample	Duration of Follow-up	Diet and nutrition	Outcome	Method
**Cross-sectional study**	TMAO	Rath et al. (2021)	425	Germany	Blood	N/A	Salad, fruits, vegetables	Carotid intima-media thickness	Linear regressions; structural equation model
			Eggs and choline were positively associated with plasma TMAO levels.						
	ImP	Molinaro et al. (2020), Senthong et al. (2016)	1,958	France	Blood	N/A	Histidine, saturated fat intake, fiber, and unsaturated fat intake	T2D	Partial correlations random forest
			Serum ImP levels were higher in participants with prediabetes and T2D. ImP was positively associated with saturated fat intake (driven by high cheese intake) and negative associated with fiber and unsaturated fat intake (driven by reduced intake of vegetables and nuts).						
	SCFAs	de la Cuesta-Zuluaga et al. (2018)	441	USA	Stool	N/A	N/A	Gut permeability, markers of metabolic dysregulation, obesity and hypertension	Regression models
			Higher SCFA concentrations were associated with a measure of gut permeability, markers of metabolic dysregulation, obesity, and hypertension.						
		Sanna et al. (2019)	952 (discovery cohort); 500,000 (validation cohort)	Netherlands	Stool	N/A	N/A	17 anthropometric and glycemic traits	Bidirectional Mendelian Randomization
			Increased fecal butyrate levels were causally associated with improved insulin response following an oral glucose test, and increased fecal propionate levels were causally associated with higher risk of T2D.						
	Bile acids	Trefflich et al. (2019)	72	Germany	Blood and stool	N/A	Vegans, fat, and fiber	N/A	Logistic regression
			Serum primary and glycine-conjugated bile acids were higher and all fecal bile acids were lower in vegans compared with omnivores; fat intake had positive and fiber intake had inverse associations with bile acids levels.						
	GDUT	Pignanelli et al. (2019)	316	Canada	Blood	N/A	Red meat, egg yolk	Reduction of eGFR	Linear regression
			Intake of meat/amino acids contributed to plasma levels of all GDUT. Intake of choline/pre-TMA, largely from egg yolk, contributed to plasma levels of TMAO, hippuric acid, and p-cresyl gluronide.						
**Prospective study**	TMAO	Wang et al. (2014)	3,903	USA	Blood	3 years	Choline and betaine	MACE	Cox proportional hazards models
			Choline and betaine were associated with TMAO. High choline and betaine levels were only associated with higher risk of MACE with concomitant increase in TMAO.						
		Senthong et al. (2016)	2,235	USA	Blood	5 years	N/A	All-cause mortality	Cox proportional hazards models
			Higher plasma TMAO levels were associated with higher long-term mortality risk among patients with stable coronary artery disease managed with optimal medical treatment.						
		Li et al. (2017)	530 (discovery cohort) 1683 (validation cohort)	USA and Swiss	Blood	Discovery: 6 months; validation: 1 year	N/A	MACE; mortality	Cox proportional hazards models
			Plasma TMAO levels among patients with chest pain were associated with both near- and long-term risks of cardiovascular events.						
		Yu et al. (2019)	550	China	Urine	N/A	N/A	Coronary heart disease	Conditional logistic regression
			Urinary TMAO was associated with risk of coronary heart disease.						
		Li et al. (2021)	307	USA	Blood	6 months	Red meat and choline	HbA1c, HDL-C	Generalized random-effects linear regressions (MaAsLin2)
			Recent intake of red meat and choline, and habitual intake of egg, dairy, and fish were associated with increased plasma TMAO levels. Associations of higher intake of red meat and choline with higher TMAO levels were only among participants who were microbial TMAO-producers.						
		Lee et al. (2021)	4,131 (incident) and 1449 (recurrent)	USA	Blood	7 years	N/A	Atherosclerotic cardiovascular disease	Cox proportional hazards models
			Serial measures of TMAO were associated with higher risk of incident atherosclerotic cardiovascular disease and recurrent atherosclerotic cardiovascular disease.						
		Wei et al. (2022)	955	China	Blood	7 years	N/A	Cardiovascular death or heart transplantation	Cox proportional hazards models
			Higher plasma TMAO levels were associated with increased risk of the composite outcome of cardiovascular death or heart transplantation. The FMO3 AA-genotype in rs2266782 was associated with lower plasma TMAO levels.						
		Wang et al. (2022)	3,931	USA	Blood	Over 12 years	Dietary Meat	Atherosclerotic cardiovascular disease	Cox proportional hazards models
			Higher meat intake was associated with incident atherosclerotic cardiovascular disease, partly mediated by l-carnitine, abundant in red meat.						
	Indole derivatives	Szabo de Edelenyi et al. (2021)	891	France	Urine	N/A	Fruit and vegetables consumption	Recurrent depressive symptoms	Conditional logistic models; general linear models
			Fruit and vegetable intakes were inversely associated with urinary 3-indoxylsulfate concentration.						
		Qi et al. (2022)	9,180	USA	Blood	Over 5 years	Fiber-rich foods; milk	T2D	Cox proportional hazards models
			Higher milk intake among lactase non-persistent participants, and higher fiber intake were associated with a favorable profile of circulating tryptophan metabolites for T2D						
	SCFAs	Neuffer et al. (2022)	418 (discovery cohort); 420 (validation cohort)	French	Blood	12 years	Meat and cheese	Cognitive decline	Logistic regression
			Higher serum propionic acid levels were associated with increased odds of cognitive decline and were positively associated with intakes of meat and cheese.						
	Bile acids	Jiang et al. (2020)	1,879	China	Stool	6.2 years	Fruit	T2D	Partial correlation analysis, logistic regression
			Fruit-related gut microbiota index was inversely associated with fecal cholic acid and 3-dehydrocholic acid, which were positively associated with T2D risk.						
		Jiang et al. (2022)	1,809	China	Stool	6 years	Habitual tea consumption	Cardiometabolic diseases	Linear regression model
			Habitual tea consumption was inversely associated with chronic insomnia-disrupted bile acid norcholic acid.						
	Enterolactone and enterodiol	Hu et al. (2015)	1,111	USA	Urine	10 years	Dietary lignans	Weight change	Linear mixed-effects model
			Higher urinary lignan metabolites, especially enterodiol, were associated with modestly slower weight gain.						
**Intervention study**	Indole derivatives	Kang et al. (2022)	20	USA	Blood	12 weeks	Prebiotic fiber supplement	N/A	Linear mixed models
			A prebiotic fiber supplement taken daily could increase the production of health-promoting bacteria-derived metabolites, such as indolepropionate in healthy individuals with a habitual low-fiber diet.						
	Imp	Nishimoto et al. (2022)	29	Japan	Stool	24 weeks	Resistant maltodextrin	N/A	Wilcoxon-Mann-Whitney test
			Resistant maltodextrin (one of the dietary fibers) could reduce virulence metabolites, such as ImP.						

TMAO is one of the most studied microbial metabolites, which is produced by the gut microbiota from foods rich in carnitine, phosphatidylcholine, and choline, such as red meat and eggs (Brown and Hazen, 2018; Dehghan et al., 2020). Leveraging longitudinal cohort from healthy US men, Li et al. showed that habitual intakes of red meat, egg, dairy, and fish were associated with increased plasma TMAO levels (Li et al., 2021). They further found that the positive association of red meat and choline with TMAO concentrations were only among participants with abundant TMAO-predicting species, suggesting the essential role of gut microbiota in TMAO production (Li et al., 2021). Consistently, a study based on the German population also showed that eggs and choline were positively associated with plasma TMAO levels (Rath et al., 2021). Furthermore, the detrimental relationships between TMAO levels and cardiovascular diseases have been manifested in many studies (Senthong et al., 2016; Li et al., 2017; Yu et al., 2019; Lee et al., 2021; Wei et al., 2022). The associations of diet and nutrient with cardiovascular diseases may depend on the TMAO levels. For example, a previous study showed that TMAO-related metabolites (TMAO, γ-butyrobetaine, and crotonobetaine) together mediated the associations of unprocessed red meat (mediated proportion: 10.6%), total meat (mediated proportion: 7.8%), and total animal-source foods (mediated proportion: 9.2%) with risk of atherosclerotic cardiovascular disease (Wang et al., 2022b). Another study demonstrated that the associations of high levels of plasma choline and betaine with major adverse cardiac events (MACE) were significant only among those with concomitant elevated TMAO levels, while neither choline nor betaine was associated with risk of MACE when adjusting for TMAO levels (Wang et al., 2014).

Tryptophan, an essential amino acid, is beneficial for human health, which can be converted to a variety of indole derivatives (e.g., indoleacetate, indolelactate, and indolepropionate) by gut microbiota (Roager and Licht, 2018). Qi et al. showed that indolelactate was positively while indolepropionate was inversely associated with risk of T2D (Qi et al., 2022). They also found that higher fiber intake was associated with higher levels of indolepropionate, and lower levels of indolelactate and indoxyl sulfate. As a support, one recent intervention study found that a prebiotic fiber supplement could significantly increase plasma indolepropionate concentrations compared to the placebo among healthy young participants consuming a diet low in fiber (Kang et al., 2022). Higher intakes of vegetables, fruits, whole grains, nuts, and legumes, and lower intakes of refined grains and red meat were associated with higher serum indolepropionate levels (Qi et al., 2022). Fruit and vegetable intakes were also inversely associated with urinary 3-indoxylsulfate concentrations (Szabo de Edelenyi et al., 2021).

Imidazole propionate (ImP) is a microbial metabolite from histidine. Serum ImP levels were higher in participants with prediabetes or T2D, and this metabolite was also associated with gut microbial enterotype and gene richness (Molinaro et al., 2020). In addition, ImP levels were positively associated with saturated fat intake (driven by high cheese intake), and negatively associated with fiber and unsaturated fat intake (driven by reduced intake of vegetables and nuts) (Molinaro et al., 2020). One intervention study conducted by Nishimoto et al. demonstrated that the intake of resistant maltodextrin (one of the dietary fibers) could reduce fecal ImP levels among Japanese participants (Nishimoto et al., 2022). For the dietary pattern, serum ImP levels were inversely associated with the alternate Healthy Eating Index, dietary diversity score, and MedDiet score (Molinaro et al., 2020). Overall, dietary fiber, unsaturated fat intake, and healthy dietary patterns may be inversely associated with T2D and glucose metabolism disorder through reducing the levels of ImP.

SCFAs, including acetic acid, propionic acid, and butyric acid are produced by microbial fermentation of dietary fiber. The evidence for the associations of SCFAs with diet and health primarily comes from rodent studies (Krautkramer et al., 2021). In human studies, Cuesta-Zuluaga et al. showed that higher fecal SCFA concentrations were associated with gut permeability, markers of metabolic dysregulation, obesity, and hypertension (de la Cuesta-Zuluaga et al., 2018). Utilizing the bidirectional Mendelian randomization analysis, Sanna et al. demonstrated that high levels of fecal butyrate was causally associated with improved insulin sensitivity, while higher levels of fecal propionate were causally associated with higher risk of T2D (Sanna et al., 2019). In addition, higher serum propionic acid levels were associated with increased risk of cognitive decline and were positively correlated with intakes of meat and cheese (Neuffer et al., 2022).

Bile acids (BAs), which are synthesized from cholesterol in the liver, act as important intermediates in gut microbiota-host crosstalk. A cross-sectional study showed that serum primary and glycine-conjugated BAs in vegans were higher but all fecal BAs were lower than omnivores (Trefflich et al., 2019). Through deriving dietary patterns, this study also showed that fat intake was positively, while fiber intake was inversely associated with BAs levels (Trefflich et al., 2019). In addition, Jiang et al. found that fruit-related gut microbiota index was inversely associated with fecal cholic acid and 3-dehydrocholic acid, which were positively associated with T2D risk (Jiang et al., 2020). Another study discovered that habitual tea consumption was inversely associated with chronic insomnia-disrupted BA norcholic acid (Jiang et al., 2022), suggesting the potential role of habitual tea consumption in treating chronic insomnia through targeting BAs. Meanwhile, Wang et al. systematically explored the associations between diet, microbial genetics, and plasma BA composition, providing a valuable resource for future studies (Wang et al., 2021b).

Except for the TMAO, there are other microbial metabolites that belong to gut-derived uremic toxins (GDUT), including p-cresyl sulfate, hippuric acid, p-cresyl glucuronide, pheny acetyl glutamine, and phenyl sulfate. Plasma levels of GDUT were all significantly higher even with moderate reduction of renal function (Pignanelli et al., 2019). Intake of meat/amino acids contributed to plasma abundances of all GDUT. Plasma levels of hippuric acid and p-cresyl gluronide were significantly explained by intakes of TMA, largely from egg yolk (Pignanelli et al., 2019). Thus, participants with impaired renal function should limit the intake of red meat, animal protein, and egg yolk. In addition, intestinal microbial metabolites of dietary lignans such as enterolactone and enterodiol were associated with modestly slower weight gain (Hu et al., 2015).

Apart from above studies, several recent studies have systematically explored the associations of diet and gut microbiome with more than 1000 metabolites (Bar et al., 2020; Chen et al., 2022), providing important resources for potential diet-related microbial metabolite investigations in future.

## Role of gut microbiome in the precision nutrition research

**Figure 2.**
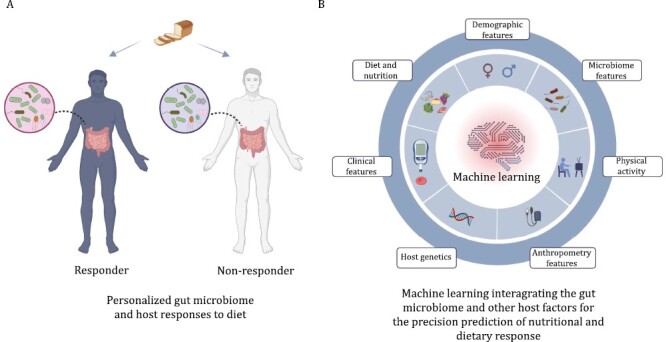
**Gut microbiome-based precision nutrition.** (A) Host responses to the diet and nutrition are highly variable across individuals. (B) Machine learning integrating the gut microbiome and other host factors could predict the personalized nutritional and dietary response. This figure was created with BioRender.com.

Precision nutrition is a broad concept to answer “What to Eat to Stay Healthy,” covering multidisciplinary integration, among which, gut microbiome is becoming an essential component (Rodgers and Collins, 2020). In the above sections, we have summarized how dietary pattern, food groups, or nutrients affect the gut microbiota diversity, taxa, and functions across different cohorts or clinical studies. Here we review another important area of gut microbiome-based precision nutrition for the identification of key microbiota features that predict the host phenotypic response to diet, which can then inform the design of microbiome-guided personalized nutrition guidelines for diverse individuals (Fig. 2 and Table 4).

**Table 4.** Literature supporting the role of gut microbiome in the precision nutrition research.

Study	Study design	Sample size	Country	Sequencing	Duration of intervention	Health outcome	Method
Hjorth et al. (2018)	Intervention study	62	Danish	PCR	26 weeks	Body weight	Linear mixed model
	Participants with Prevotella-dominate microbial features lost more body weight on the high-fiber diet intervention than the western diet intervention, whereas no difference in body weight was observed for the participants with Bacteroides-dominate microbial features.						
Hjorth et al. (2019)	Intervention study	52	Danish	16S rRNA	24 weeks	Body weight	Linear mixed model
	Participants with a high Prevotella-to-Bacteroides ratio are more susceptible to weight loss on a diet rich in fiber.						
Cotillard et al. (2013)	Intervention study	49	France	Metagenomics	12 weeks	HOMA-IR, Triglycerides and hsCRP	Logistic regression
	Weight-maintenance diet intervention on the clinical phenotypes was less pronounced for participants with lower microbial gene richness.						
Zeevi et al. (2015)	Intervention study	800 (discovery cohort); 100 (validation cohort); 26 (randomised controlled trial)	Israel	16S rRNA, Metagenomics	1 week	PPGRs	Gradient boosting regression
	PPGRs were highly variable across individuals. Personalized PPGRs could be predicted by clinical and gut microbial features. Short-term personalized dietary interventions based on the machine learning prediction successfully lower PPGRs.						
Ben-Yacov et al. (2021)	Intervention study	225 participants with prediabetes	Israel	Metagenomics	6-month intervention	Daily time of glucose levels >140 mg/dL (7.8 mmol/L), OGTT and HbA1c	Gradient boosting regression
	A machine learning-predicted personalized postprandial-targeting diet improved glycemic control more effectively than a MedDiet.						
Mendes-Soares et al. (2019)	Intervention study	327	USA	Metagenomics	1 week	Postprandial glycemic response	Gradient boosting regression
	The modeling framework described in Zeevi et al. for an Israeli cohort could be applicable to a Midwestern population.						
Berry et al. (2020)	Intervention study	1,002 (discovery cohort);100 (validation cohort)	UK and USA	16S rRNA	2 weeks	Postprandial triglyceride, glucose, and insulin responses.	Random forest regression
	Large inter-individual variability in postprandial responses of blood triglyceride (103%), glucose (68%) and insulin (59%) were observed. Machine-learning model based on the gut microbiome and clinical factors could predicted both triglyceride (*r* = 0.47) and glycemic (*r* = 0.77) responses to food intake.						
Bennet et al. (2018)	Intervention study	67	Sweden	16S rRNA	4 weeks	IBS symptom severity	OPLS-DA
	Low fermentable oligosaccharides, disaccharides, monosaccharides, and polyols, but not traditional IBS diet responders could be discriminated from non-responders before the intervention based on faecal bacterial profiles.						
Korem et al. (2017)	Intervention study	20	Israel	16S rRNA, Metagenomics	2 weeks	PPGRs	Gradient boosting regression
	Glycemic response to different bread types varies greatly across people. The type of bread that induces the lower glycemic response in each person can be predicted based on the gut microbial features.						
Deehan et al. (2020)	Intervention study	40	Canada	16S rRNA	4 weeks	Fecal microbiota composition, SCFA profiles, and perceived GI tolerance	2-way ANOVA, GEE
	Small differences in dietary fibers structure yet distinct effects on the gut microbiome composition that are linked to directed shifts in the output of either propionate or butyrate.						
Suez et al. (2022)	Intervention study	120	Israel	Metagenomics	4 weeks	Stool and oral microbiome, plasma metabolome, and glucose tolerance	Linear mixed models, PERMANOV
	Non-nutritive sweeteners personalized altered stool and oral microbiome and plasma metabolome. Only saccharin and sucralose causally linked to impaired glycemic responses						

PPGRs, postprandial glycemic response.

Similar as the application of nutrigenetics in the precision nutrition, a number of studies have consistently found that stratifying individuals according to gut microbial enterotypes (dominance of either *Prevotella* or *Bacteroides*) is applicable, which is the initial application of gut microbiome-based precision nutrition research (Hjorth et al., 2018; Hjorth et al., 2019). For example, a 26-week randomized controlled trial showed that participants with *Prevotella*-dominated microbial enterotype lost more body weight on a high-fiber diet intervention than a western diet intervention, whereas no difference in body weight change was observed for the participants with *Bacteroides*-dominated microbial enterotype (Hjorth et al., 2018). Consistently, another 24-week dietary intervention study found that participants with high *Prevotella*-to-*Bacteroides* ratio lost more body weight and body fat compared to participants with low *Prevotella*-to-*Bacteroides* ratio (Hjorth et al., 2019). Participants with a high *Prevotella*-to-*Bacteroides* ratio might result in improvement of enzymatic capacity for fiber digestion and glucose metabolism (Hjorth et al., 2019). Microbial gene richness is another important microbial index for the subgroup-based precision nutrition. A 12-week weight-maintenance diet intervention study included 38 obese and 11 overweight participants, and found that the effect of weight-maintenance dietary intervention on the clinical phenotypes was less pronounced among participants with lower gut microbial gene richness (Cotillard et al., 2013).

Gut microbiome is a major factor determining the variation of individual’s metabolic response to diet, which provides a rationale to combine the gut microbial features and other host phenotypes to predict the host response for similar or different dietary challenges. A landmark study within this field was the Israeli personalized nutrition study (*n* = 800) (Zeevi et al., 2015). They found that the combination of gut microbiome, dietary factors, anthropometrics, and clinical parameters could accurately predict personalized postprandial glycemic response to real-life meals. Furthermore, 1-week personalized dietary interventions based on the prediction showed improvement in glucose metabolism. As a validation of this study, Ben-Yacov et al. randomly assigned 225 adults with prediabetes to follow a MedDiet or a machine learning-predicted personalized postprandial-targeting (PPT) diet for a 6-month intervention and additional 6-month follow-up (Ben-Yacov et al., 2021). They found that a PPT diet improved glycemic control more effectively than a MedDiet (Ben-Yacov et al., 2021). With the same study design and predictive model as the Israeli study, Mendes-Soares et al. enrolled 327 participants without diabetes from the USA. They found that the postprandial response to the same foods varied across participants, and the modeling framework developed by the Israeli study could be applicable to a Midwestern population (Mendes-Soares et al., 2019). Inspired by the Israeli personalized nutrition study, a large-scale twin study recruited more than 1000 healthy adults from the UK to USA and undertook a 2-week interventional trial. They observed that even genetically similar twins had differently postprandial responses of blood triglycerides and glucose to identical meals. They highlighted that environmental factors, mainly gut microbiome could predict the triglycerides and glycemic responses to diet (Berry et al., 2020).

For a specific disease population, like inflammatory bowel syndrome (IBS) patients, a 4 weeks intervention study included 67 participants who were randomized to traditional IBS (*n* = 34) or low fermentable oligosaccharides, disaccharides, monosaccharides, and polyols (FODMAPs) (*n* = 33) diets (Bennet et al., 2018). They found that low FODMAPs, but not traditional IBS diet responders could be discriminated from non-responders based on gut microbiome before the intervention (Bennet et al., 2018).

Indeed, even for the specific food groups or nutrients, their different subtypes may have personalized effects on the human metabolism, which could be predicted by gut microbiome. Korem et al. performed a crossover clinical trial for the consumption of industrially made white bread or artisanal sourdough-leavened whole-grain bread (Korem et al., 2017), and found that glycemic response to different bread types varies greatly across individuals, and the type of bread that induces the lower glycemic response in each person can be predicted based on the gut microbial features (Korem et al., 2017). Deehan et al. showed that small differences in dietary fiber structure could have distinct effects on the gut microbiome composition, leading to directed shifts in the output of either propionate or butyrate (Deehan et al., 2020). Suez et al. found that different type of non-nutritive sweeteners personalized altered stool and oral microbiome. Only saccharin and sucralose were causally linked to impaired glycemic responses (Suez et al., 2022).

## Conclusions and future perspectives

In this review, we have discussed the latest advances in understanding the role of the microbiome in nutrition and human health. Microbiome-based nutritional (i.e., nutri-microbiome) epidemiological studies have uncovered many microbes and microbial metabolomic targets for dietary intervention and disease prevention. Furthermore, the integration of nutrition with microbiome provides new approach for understanding the mechanism behind the diet-disease associations. In particular, inter-individual differences in postprandial metabolic responses to diet challenge the logic of “one-size-fits-all” dietary recommendations. Gut microbiome have been demonstrated as one of the effective predictors of host response to a particular diet. Gut microbiome-based precision nutrition provides hope for further advancements in control and treatment of disease at individual level.

The advances in nutri-microbiome epidemiological studies are promising, yet, there are several limitations in this field, including study design, technologies, analysis methods, and results interpretation. First, the nutrition and microbiome associations are generally studied in cross-sectional or short-term longitudinal settings, while the human gut hosts a dynamic microbial ecosystem which is continuously perturbed by daily dietary intake (Johnson et al., 2019; Olsson et al., 2022). Therefore, well-designed longitudinal investigations with repeat measures, ideally randomized controlled trials, are needed to assess diet-gut microbiota interaction on the human health. Second, geographic location, customs, and culture, seasonal variations in food availability could all impact the dietary choices and the gut microbiome heterogeneity (Ecklu-Mensah et al., 2022), and therefore caution should be taken in extrapolating findings from different geographic location or ethnic groups. Third, mass spectrometry metabolomic profiling could detect thousands of unique signal features. However, it is currently difficult to accurately identify metabolites for many of these signal features, which affects the discovery of new metabolomic biomarkers. Finally, machine learning has been widely and effectively applied in the precision nutrition studies, while its prediction algorithms are usually difficult to interpret.

Despite several limitations and obstacles, recent advances in the field bode well for the future. The repeated-crossover design of an n-of-1 trial is an advantageous approach to compare the effect of two or more interventions for an individual (Gabler et al., 2011; Potter et al., 2021). Nutritional n-of-1 clinical trials, in the context of the human gut microbiota provide a unique opportunity to characterize the host response and gut microbial variability of nutritional interventions at the individual level (Zheng and Ordovás, 2021). Mobile apps and wearable devices facilitate the real-time assessment of dietary intake, nutritional biomarker variations, and changes of health-related vital signs (Sempionatto et al., 2021). Current advances in wearable devices have proved prerequisite for precision nutrition (Sempionatto et al., 2021; Merino et al., 2022). Furthermore, interpretable machine learning has received more and more attentions in recent years (Murdoch et al., 2019). By integrating cutting-edge wearable and mobile sensing technologies with interpretable machine learning, precision nutrition is expected to provide effective personalized nutrition guidance and interventions. The applications of metabolomics in the field of nutrition and microbiome could greatly benefit from the improvement of the stability and repeatability of metabolomic profiling, the standardization of analytical methods and strategies, the advances in metabolic peak identification, the comprehensive utilization of different biological samples for metabolomic profiling. Moreover, research on the interactions of the gut multi-kingdom ecosystem (including bacteria, virome, and mycobiome community, etc.) with diet and nutrition for human health is an emerging field of great interest.

To conclude, systematic integration of microbiome with the large-scale nutritional epidemiological cohort studies fosters the development of nutri-microbiome epidemiology, a rising field to disentangle the relationship among diet, nutrition, microbiome, and human health. These new progresses stimulate the further development of microbiome-based precision nutrition research.

## Data Availability

No dataset was generated or analyzed during this study.

## Abbreviations

BAs: bile acids
FODMAPs: fermentable oligosaccharides, disaccharides, monosaccharides, and polyols
GDUT: gut-derived uremic toxins
GL: glycemic load
IBS: inflammatory bowel syndrome
ImP: imidazole propionate
MACE: major adverse cardiac events
MedDiet: Mediterranean diet
PPT: personalized postprandial-targeting
PUFAs: polyunsaturated fatty acids
SCFA: short-chain fatty acids
TMAO: trimethylamine N-oxide;
T2D: , Type 2 diabetes

## References

[CIT0001] Ang QY , Alexander M, Newman JC et al. Ketogenic diets alter the gut microbiome resulting in decreased intestinal Th17 cells. Cell 2020;181:1263–1275.e16. 32437658 10.1016/j.cell.2020.04.027 PMC7293577

[CIT0002] Asnicar F , Berry SE, Valdes AM et al. Microbiome connections with host metabolism and habitual diet from 1,098 deeply phenotyped individuals. Nat Med 2021;12:13. 10.1038/s41591-020-01183-8 PMC8353542 33432175

[CIT0003] Bar N , Korem T, Weissbrod O et al. A reference map of potential determinants for the human serum metabolome. Nature 2020;588:135–140. 33177712 10.1038/s41586-020-2896-2

[CIT0004] Bennet SMP , Böhn L, Störsrud S et al. Multivariate modelling of faecal bacterial profiles of patients with IBS predicts responsiveness to a diet low in FODMAPs. Gut 2018;67:872–881. 28416515 10.1136/gutjnl-2016-313128

[CIT0005] Ben-Yacov O , Godneva A, Rein M et al. Personalized postprandial glucose response-targeting diet versus Mediterranean diet for glycemic control in prediabetes. Diabetes Care 2021;44:1980–1991. 34301736 10.2337/dc21-0162

[CIT0006] Berry SE , Valdes AM, Drew DA et al. Human postprandial responses to food and potential for precision nutrition. Nat Med 2020;26:964–973. 32528151 10.1038/s41591-020-0934-0 PMC8265154

[CIT0007] Bolte LA , Vich Vila A, Imhann F et al. Long-term dietary patterns are associated with pro-inflammatory and anti-inflammatory features of the gut microbiome. Gut 2021;70:1–12. 10.1136/gutjnl-2020-322670 PMC8223641 33811041

[CIT0008] Borodulin K , Tolonen H, Jousilahti P et al. Cohort profile: the National FINRISK Study. Int J Epidemiol 2018;47:696–696i. 29165699 10.1093/ije/dyx239

[CIT0009] Boronat A , Rodriguez-Morató J, Serreli G et al. Contribution of biotransformations carried out by the microbiota, drug-metabolizing enzymes, and transport proteins to the biological activities of phytochemicals found in the diet. Adv Nutr 2021;12:2172–2189. 34388248 10.1093/advances/nmab085 PMC8634308

[CIT0010] Breuninger TA , Wawro N, Breuninger J et al. Associations between habitual diet, metabolic disease, and the gut microbiota using latent Dirichlet allocation. Microbiome 2021;9:1–18. 33726846 10.1186/s40168-020-00969-9 PMC7967986

[CIT0011] Brown JM , Hazen SL. Microbial modulation of cardiovascular disease. Nat Rev Microbiol 2018;16:171–181. 29307889 10.1038/nrmicro.2017.149 PMC5885760

[CIT0012] Chassaing B , Koren O, Goodrich JK et al. Dietary emulsifiers impact the mouse gut microbiota promoting colitis and metabolic syndrome. Nature 2015;519:92–96. 25731162 10.1038/nature14232 PMC4910713

[CIT0013] Chen L , Zhernakova DV, Kurilshikov A et al. Influence of the microbiome, diet and genetics on inter-individual variation in the human plasma metabolome. Nat Med 2022;28:2333–2343. 36216932 10.1038/s41591-022-02014-8 PMC9671809

[CIT0014] Costantini L , Molinari R, Farinon B et al. Impact of Omega-3 fatty acids on the gut microbiota. Int J Mol Sci 2017;18:2645. 29215589 10.3390/ijms18122645 PMC5751248

[CIT0015] Cotillard A , Kennedy SP, Kong LC et al. Dietary intervention impact on gut microbial gene richness. Nature 2013;500:585–588. 23985875 10.1038/nature12480

[CIT0016] Cuevas-Sierra A , Milagro FI, Aranaz P et al. Gut microbiota differences according to ultra-processed food consumption in a Spanish population. Nutrients 2021;13:2710. 34444870 10.3390/nu13082710 PMC8398738

[CIT0017] Dall’Asta M , Laghi L, Morselli S et al. Pre-pregnancy diet and vaginal environment in caucasian pregnant women: an exploratory study. Front Mol Biosci 2021;8:702370. 34395531 10.3389/fmolb.2021.702370 PMC8356051

[CIT0018] Dang AT , Marsland BJ. Microbes, metabolites, and the gut–lung axis. Mucosal Immunol 2019;12:843–850. 30976087 10.1038/s41385-019-0160-6

[CIT0019] Deehan EC , Yang C, Perez-Muñoz ME et al. Precision microbiome modulation with discrete dietary fiber structures directs short-chain fatty acid production. Cell Host Microbe 2020;27:389–404.e6. 32004499 10.1016/j.chom.2020.01.006

[CIT0020] De Filippis F , Pellegrini N, Vannini L et al. High-level adherence to a Mediterranean diet beneficially impacts the gut microbiota and associated metabolome. Gut 2016;65:1812–1821. 26416813 10.1136/gutjnl-2015-309957

[CIT0021] Dehghan P , Farhangi MA, Nikniaz L et al. Gut microbiota-derived metabolite trimethylamine N-oxide (TMAO) potentially increases the risk of obesity in adults: an exploratory systematic review and dose-response meta- analysis. Obes Rev 2020;21:e12993. 32017391 10.1111/obr.12993

[CIT0022] Deleu S , Machiels K, Raes J et al. Short chain fatty acids and its producing organisms: an overlooked therapy for IBD? EBioMedicine 2021;66:103293. 33813134 10.1016/j.ebiom.2021.103293 PMC8047503

[CIT0023] de la Cuesta-Zuluaga J , Mueller NT, Álvarez-Quintero R et al. Higher fecal short-chain fatty acid levels are associated with gut microbiome dysbiosis, obesity, hypertension and cardiometabolic disease risk factors. Nutrients 2018;11:1–16. 30591685 10.3390/nu11010051 PMC6356834

[CIT0024] Ecklu-Mensah G , Gilbert J, Devkota S. Dietary selection pressures and their impact on the gut microbiome. Cell Mol Gastroenterol Hepatol 2022;13:7–18. 34329765 10.1016/j.jcmgh.2021.07.009 PMC8600059

[CIT0025] Esberg A , Eriksson L, Hasslöf P et al. Using oral microbiota data to design a short sucrose intake index. Nutrients 2021;13:1400. 33919427 10.3390/nu13051400 PMC8143301

[CIT0026] Fan Y , Pedersen O. Gut microbiota in human metabolic health and disease. Nat Rev Microbiol 2021;19:55–71. 32887946 10.1038/s41579-020-0433-9

[CIT0027] Faria A , Fernandes I, Norberto S et al. Interplay between anthocyanins and gut microbiota. J Agric Food Chem 2014;62:6898–6902. 24915058 10.1021/jf501808a

[CIT0028] France M , Alizadeh M, Brown S et al. Towards a deeper understanding of the vaginal microbiota. Nat Microbiol 2022;7:367–378. 35246662 10.1038/s41564-022-01083-2 PMC8910585

[CIT0029] Gabler NB , Duan N, Vohra S et al. N-of-1 trials in the medical literature: a systematic review. Med Care 2011;49:761–768. 21478771 10.1097/MLR.0b013e318215d90d

[CIT0030] Garcia-Mantrana I , Selma-Royo M, Alcantara C et al. Shifts on gut microbiota associated to Mediterranean diet adherence and specific dietary intakes on general adult population. Front Microbiol 2018;9:890. 29867803 10.3389/fmicb.2018.00890 PMC5949328

[CIT0031] Ghosh TS , Rampelli S, Jeffery B et al. Mediterranean diet intervention alters the gut microbiome in older people reducing frailty and improving health status: the NU-AGE 1-year dietary intervention across five European countries. Gut 2020;69:1218–1228. 32066625 10.1136/gutjnl-2019-319654 PMC7306987

[CIT0032] Gou W , Ling C-W, He Y et al. Interpretable machine learning framework reveals robust gut microbiome features associated with Type 2 diabetes. Diabetes Care 2021;44:358–366. 33288652 10.2337/dc20-1536 PMC7818326

[CIT0033] Gou W , Ling C-W, He Y et al. Westlake gut project: a consortium of microbiome epidemiology for the gut microbiome and health research in China. Med Microecol 2022;14:100064.

[CIT0034] He J , Zhang P, Shen L et al. Short-chain fatty acids and their association with signalling pathways in inflammation, glucose and lipid metabolism. Int J Mol Sci 2020;21:6356. 32887215 10.3390/ijms21176356 PMC7503625

[CIT0035] Hjorth﻿ MF, Roager﻿ HM, Larsen﻿ TM et al. Pre-treatment microbial Prevotella-to-Bacteroides ratio, determines body fat loss success during a 6-month randomized controlled diet intervention. Int J Obes (Lond) 2018;42:580–583. 28883543 10.1038/ijo.2017.220 PMC5880576

[CIT0036] Hjorth﻿ MF, Blædel﻿ T, Bendtsen﻿ LQ et al. Prevotella-to-Bacteroides ratio predicts body weight and fat loss success on 24-week diets varying in macronutrient composition and dietary fiber: results from a post-hoc analysis. Int J Obes (Lond) 2019;43:149–157. 29777234 10.1038/s41366-018-0093-2 PMC6331389

[CIT0037] Hu Y , Song Y, Franke AA et al. A prospective investigation of the association between urinary excretion of dietary lignan metabolites and weight change in US women. Am J Epidemiol 2015;182:503–511. 26290574 10.1093/aje/kwv091 PMC4580533

[CIT0038] Huang X , Gao Y, Chen W et al. Dietary variety relates to gut microbiota diversity and abundance in humans. Eur J Nutr 2022;61:3915–3928. 35764724 10.1007/s00394-022-02929-5

[CIT0039] Jiang Z , Sun T, He Y et al. Dietary fruit and vegetable intake, gut microbiota, and type 2 diabetes: results from two large human cohort studies. BMC Med 2020;18:1–11. 33267887 10.1186/s12916-020-01842-0 PMC7712977

[CIT0040] Jiang Z , Zhuo L, He Y et al. The gut microbiota-bile acid axis links the positive association between chronic insomnia and cardiometabolic diseases. Nat Commun 2022;13:3002. 35637254 10.1038/s41467-022-30712-x PMC9151781

[CIT0041] Johnson AJ , Vangay P, Al-Ghalith GA et al. Daily sampling reveals personalized diet-microbiome associations in humans. Cell Host Microbe 2019;25:789–802.e5. 31194939 10.1016/j.chom.2019.05.005

[CIT0042] Kang JW , Tang X, Walton CJ et al. Multi-omic analyses reveal bifidogenic effect and metabolomic shifts in healthy human cohort supplemented with a prebiotic dietary fiber blend. Front Nutr 2022;9:908534. 35782954 10.3389/fnut.2022.908534 PMC9248813

[CIT0043] Kim H , Caulfield LE, Garcia-Larsen V et al. Plant-based diets are associated with a lower risk of incident cardiovascular disease, cardiovascular disease mortality, and all-cause mortality in a general population of middle-aged adults. J Am Heart Assoc 2019;8:e012865. 31387433 10.1161/JAHA.119.012865 PMC6759882

[CIT0044] Koeth RA , Wang Z, Levison BS et al. Intestinal microbiota metabolism of L-carnitine, a nutrient in red meat, promotes atherosclerosis. Nat Med 2013;19:576–585. 23563705 10.1038/nm.3145 PMC3650111

[CIT0045] Kolodziejczyk AA , Zheng D, Elinav E. Diet-microbiota interactions and personalized nutrition. Nat Rev Microbiol 2019;17:742–753. 31541197 10.1038/s41579-019-0256-8

[CIT0046] Korem T , Zeevi D, Zmora N et al. Bread affects clinical parameters and induces gut microbiome-associated personal glycemic responses. Cell Metab 2017;25:1243–1253.e5. 28591632 10.1016/j.cmet.2017.05.002

[CIT0047] Krautkramer KA , Fan J, Bäckhed F. Gut microbial metabolites as multi-kingdom intermediates. Nat Rev Microbiol 2021;19:77–94. 32968241 10.1038/s41579-020-0438-4

[CIT0048] Lam KC , Araya RE, Huang A et al. Microbiota triggers STING-type I IFN-dependent monocyte reprogramming of the tumor microenvironment. Cell 2021;184:5338–5356.e21. 34624222 10.1016/j.cell.2021.09.019 PMC8650838

[CIT0049] Lamont RJ , Koo H, Hajishengallis G. The oral microbiota: dynamic communities and host interactions. Nat Rev Microbiol 2018;16:745–759. 30301974 10.1038/s41579-018-0089-x PMC6278837

[CIT0050] Lee Y , Nemet I, Wang Z et al. Longitudinal plasma measures of trimethylamine N-Oxide and risk of atherosclerotic cardiovascular disease events in community-based older adults. J Am Heart Assoc 2021;10:e020646. 34398665 10.1161/JAHA.120.020646 PMC8649305

[CIT0051] LeMay-Nedjelski L , Asbury MR, Butcher J et al. Maternal diet and infant feeding practices are associated with variation in the human milk microbiota at 3 months postpartum in a cohort of women with high rates of gestational glucose intolerance. J Nutr 2021;151:320–329. 32886107 10.1093/jn/nxaa248 PMC7850034

[CIT0052] Li XS , Obeid S, Klingenberg R et al. Gut microbiota-dependent trimethylamine N-oxide in acute coronary syndromes: a prognostic marker for incident cardiovascular events beyond traditional risk factors. Eur Heart J 2017;38:814–824. 28077467 10.1093/eurheartj/ehw582 PMC5837488

[CIT0054] Li J , Li Y, Ivey KL et al. Interplay between diet and gut microbiome, and circulating concentrations of trimethylamine N-oxide: findings from a longitudinal cohort of US men. Gut 2022;71:724–733. 33926968 10.1136/gutjnl-2020-322473 PMC8553812

[CIT0055] Liu Y , Ajami NJ, El-Serag HB et al. Dietary quality and the colonic mucosa–associated gut microbiome in humans. Am J Clin Nutr 2019;110:701. 31291462 10.1093/ajcn/nqz139 PMC6736447

[CIT0056] Liu B , Zhao J, Liu Y et al. Diversity and temporal dynamics of breast milk microbiome and its influencing factors in Chinese women during the first 6 months postpartum. Front Microbiol 2022;13:1016759. 36439858 10.3389/fmicb.2022.1016759 PMC9691949

[CIT0057] Lommi S , Manzoor M, Engberg E et al. The composition and functional capacities of saliva microbiota differ between children with low and high sweet treat consumption. Front Nutr 2022;9:864687. 35558746 10.3389/fnut.2022.864687 PMC9085455

[CIT0058] Losasso C , Eckert EM, Mastrorilli E et al. Assessing the influence of vegan, vegetarian and omnivore oriented westernized dietary styles on human gut microbiota: a cross sectional study. Front Microbiol 2018;9:317. 29556222 10.3389/fmicb.2018.00317 PMC5844980

[CIT0059] Ma Y , Fu Y, Tian Y et al. Individual postprandial glycemic responses to diet in n-of-1 trials: Westlake N-of-1 trials for macronutrient intake (WE-MACNUTR). J Nutr 2021;151:3158–3167. 34255080 10.1093/jn/nxab227 PMC8485912

[CIT0060] Mardinoglu A , Wu H, Bjornson E et al. An integrated understanding of the rapid metabolic benefits of a carbohydrate-restricted diet on hepatic steatosis in humans. Cell Metab 2018;27:559–571.e5. 29456073 10.1016/j.cmet.2018.01.005 PMC6706084

[CIT0061] McDonald D , Hyde E, Debelius JW et al. American gut: an open platform for citizen science microbiome research. MSystems 2018;3:e00031-18. 29795809 10.1128/mSystems.00031-18 PMC5954204

[CIT0062] Mendes-Soares H , Raveh-Sadka T, Azulay S et al. Model of personalized postprandial glycemic response to food developed for an Israeli cohort predicts responses in Midwestern American individuals. Am J Clin Nutr 2019;110:63–75. 31095300 10.1093/ajcn/nqz028 PMC6599737

[CIT0063] Menni C , Zierer J, Pallister T et al. Omega-3 fatty acids correlate with gut microbiome diversity and production of N-carbamylglutamate in middle aged and elderly women. Sci Rep 2017;7:11079. 28894110 10.1038/s41598-017-10382-2 PMC5593975

[CIT0064] Menni C , Louca P, Berry SE et al. High intake of vegetables is linked to lower white blood cell profile and the effect is mediated by the gut microbiome. BMC Med 2021;19:1–10. 33568158 10.1186/s12916-021-01913-w PMC7875684

[CIT0065] Merino J , Linenberg I, Bermingham KM et al. Validity of continuous glucose monitoring for categorizing glycemic responses to diet: implications for use in personalized nutrition. Am J Clin Nutr 2022;115:1569–1576. 35134821 10.1093/ajcn/nqac026 PMC9170468

[CIT0066] Merra G , Noce A, Marrone G et al. Influence of mediterranean diet on human gut microbiota. Nutrients 2021;13:1–12. 10.3390/nu13010007 PMC7822000 33375042

[CIT0067] Miao Z , Lin J-S, Mao Y et al. Erythrocyte n-6 polyunsaturated fatty acids, gut microbiota, and incident Type 2 diabetes: a prospective cohort study. 2020;43:2435–2443. 10.2337/dc20-0631 PMC7510039 32723842

[CIT0068] Miao Z , Du W, Xiao C et al. Gut microbiota signatures of long-term and short-term plant-based dietary pattern and cardiometabolic health: a prospective cohort study. BMC Med 2022;20:1–15. 35701845 10.1186/s12916-022-02402-4 PMC9199182

[CIT0132] Miao Z , Chen GD, Huo S et.al. Interaction of n-3 polyunsaturated fatty acids with host CD36 genetic variant for gut microbiome and blood lipids in human cohorts. Clin Nutr 2022;41:1724–1734. 35777111 10.1016/j.clnu.2022.05.021

[CIT0069] Millen AE , Dahhan R, Freudenheim JL et al. Dietary carbohydrate intake is associated with the subgingival plaque oral microbiome abundance and diversity in a cohort of postmenopausal women. Sci Rep 2022;12:2643. 35173205 10.1038/s41598-022-06421-2 PMC8850494

[CIT0070] Mitsou EK , Kakali A, Antonopoulou S et al. Adherence to the Mediterranean diet is associated with the gut microbiota pattern and gastrointestinal characteristics in an adult population. Br J Nutr 2017;117:1645–1655. 28789729 10.1017/S0007114517001593

[CIT0071] Molina-Montes E , Salamanca-Fernández E, Garcia-Villanova B et al. The impact of plant-based dietary patterns on cancer-related outcomes: a rapid review and meta-analysis. Nutrients 2020;12:20101–20131. 10.3390/nu12072010 PMC7400843 32640737

[CIT0072] Molinaro A , Bel Lassen P, Henricsson M et al. Imidazole propionate is increased in diabetes and associated with dietary patterns and altered microbial ecology. Nat Commun 2020;11:5881. 33208748 10.1038/s41467-020-19589-w PMC7676231

[CIT0073] Moreno-Indias I , Sánchez-Alcoholado L, Pérez-Martínez P et al. Red wine polyphenols modulate fecal microbiota and reduce markers of the metabolic syndrome in obese patients. Food Funct 2016;7:1775–1787. 26599039 10.1039/c5fo00886g

[CIT0074] Murdoch WJ , Singh C, Kumbier K et al. Definitions, methods, and applications in interpretable machine learning. Proc Natl Acad Sci USA 2019;116:22071–22080. 31619572 10.1073/pnas.1900654116 PMC6825274

[CIT0075] Naimi S , Viennois E, Gewirtz AT et al. Direct impact of commonly used dietary emulsifiers on human gut microbiota. Microbiome 2021;9:66. 33752754 10.1186/s40168-020-00996-6 PMC7986288

[CIT0076] Neuffer J , González-Domínguez R, Lefèvre-Arbogast S et al. Exploration of the gut-brain axis through metabolomics identifies serum propionic acid associated with higher cognitive decline in older persons. Nutrients 2022;14:4688. 36364950 10.3390/nu14214688 PMC9655149

[CIT0077] Nishimoto Y , Mizuguchi Y, Mori Y et al. Resistant maltodextrin intake reduces virulent metabolites in the gut environment: a randomized control study in a Japanese cohort. Front Microbiol 2022;13:644146. 35602030 10.3389/fmicb.2022.644146 PMC9116438

[CIT0078] Nordlund E , Aura A-M, Mattila I et al. Formation of phenolic microbial metabolites and short-chain fatty acids from rye, wheat, and oat bran and their fractions in the metabolical in vitro colon model. J Agric Food Chem 2012;60:8134–8145. 22731123 10.1021/jf3008037

[CIT0079] Olsson LM , Boulund F, Nilsson S et al. Dynamics of the normal gut microbiota: a longitudinal one-year population study in Sweden. Cell Host Microbe 2022;30:726–739.e3. 35349787 10.1016/j.chom.2022.03.002

[CIT0080] Peters BA , McCullough ML, Purdue MP et al. Association of coffee and tea intake with the oral microbiome: results from a large cross-sectional study. Cancer Epidemiol Biomarkers Prev 2018;27:814–821. 29703763 10.1158/1055-9965.EPI-18-0184 PMC6889868

[CIT0081] Pignanelli M , Bogiatzi C, Gloor G et al. Moderate renal impairment and toxic metabolites produced by the intestinal microbiome: dietary implications. J Ren Nutr 2019;29:55–64. 30100156 10.1053/j.jrn.2018.05.007

[CIT0082] Potter T , Vieira R, de Roos B. Perspective: application of N-of-1 methods in personalized nutrition research. Adv Nutr 2021;12:579–589. 33460438 10.1093/advances/nmaa173 PMC8166550

[CIT0083] Qi Q , Li J, Yu B et al. Host and gut microbial tryptophan metabolism and type 2 diabetes: an integrative analysis of host genetics, diet, gut microbiome and circulating metabolites in cohort studies. Gut 2022;71:1095–1105. 34127525 10.1136/gutjnl-2021-324053 PMC8697256

[CIT0084] Qian F , Liu G, Hu FB et al. Association between plant-based dietary patterns and risk of type 2 diabetes: a systematic review and meta-analysis. JAMA Intern Med 2019;179:1335–1344. 31329220 10.1001/jamainternmed.2019.2195 PMC6646993

[CIT0085] Queipo-Ortuño MI , Boto-Ordóñez M, Murri M et al. Influence of red wine polyphenols and ethanol on the gut microbiota ecology and biochemical biomarkers. Am J Clin Nutr 2012;95:1323–1334. 22552027 10.3945/ajcn.111.027847

[CIT0086] Rath S , Rox K, Kleine Bardenhorst S et al. Higher Trimethylamine-N-Oxide plasma levels with increasing age are mediated by diet and trimethylamine-forming bacteria. MSystems 2021;6:e0094521. 34519520 10.1128/mSystems.00945-21 PMC8547441

[CIT0087] Ren Z , Shi Y, Xu S et al. Gut bacteria selectively promoted by dietary fibers alleviate type 2 diabetes. Science 2018;359:1151–1156. 29590046 10.1126/science.aao5774

[CIT0088] Rinott E , Meir AY, Tsaban G et al. The effects of the Green-Mediterranean diet on cardiometabolic health are linked to gut microbiome modifications: a randomized controlled trial. Genome Med 2022;14:29. 35264213 10.1186/s13073-022-01015-z PMC8908597

[CIT0089] Roager HM , Licht TR. Microbial tryptophan catabolites in health and disease. Nat Commun 2018;9:3294. 30120222 10.1038/s41467-018-05470-4 PMC6098093

[CIT0090] Rodgers GP , Collins FS. Precision nutrition-the answer to “What to Eat to Stay Healthy”. JAMA 2020;324:735–736. 32766768 10.1001/jama.2020.13601

[CIT0091] Rosen EM , Martin CL, Siega-Riz AM et al. Is prenatal diet associated with the composition of the vaginal microbiome? Paediatr Perinat Epidemiol 2022;36:243–253. 34841560 10.1111/ppe.12830 PMC8881389

[CIT0092] Sánchez-Patán F , Cueva C, Monagas M et al. In vitro fermentation of a red wine extract by human gut microbiota: changes in microbial groups and formation of phenolic metabolites. J Agric Food Chem 2012;60:2136–2147. 22313337 10.1021/jf2040115

[CIT0093] Sanna S , van Zuydam NR, Mahajan A et al. Causal relationships among the gut microbiome, short-chain fatty acids and metabolic diseases. Nat Genet 2019;51:600–605. 30778224 10.1038/s41588-019-0350-x PMC6441384

[CIT0094] Sempionatto JR , Montiel VR-V, Vargas E et al. Wearable and mobile sensors for personalized nutrition. ACS Sensors 2021;6:1745–1760. 34008960 10.1021/acssensors.1c00553

[CIT0095] Sender R , Fuchs S, Milo R. Revised estimates for the number of human and bacteria cells in the body. PLoS Biol 2016;14:e1002533. 27541692 10.1371/journal.pbio.1002533 PMC4991899

[CIT0096] Senthong V , Wang Z, Li XS et al. Intestinal microbiota-generated metabolite Trimethylamine-N-Oxide and 5-year mortality risk in stable coronary artery disease: the contributory role of intestinal microbiota in a COURAGE-like patient cohort. J Am Heart Assoc 2016;5:e002816. 27287696 10.1161/JAHA.115.002816 PMC4937244

[CIT0097] Shaalan A , Lee S, Feart C et al. Alterations in the oral microbiome associated with diabetes, overweight, and dietary components. Front Nutr 2022;9:914715. 35873415 10.3389/fnut.2022.914715 PMC9298547

[CIT0098] Shankar Ghosh T , Rampelli S, Jeffery B et al. Gut microbiota Mediterranean diet intervention alters the gut microbiome in older people reducing frailty and improving health status: the NU-AGE 1-year dietary intervention across five European countries. Gut 2020:1–11. 10.1136/gutjnl-2019-319654 PMC7306987 32066625

[CIT0099] Shuai M , Zuo LSY, Miao Z et al. Multi-omics analyses reveal relationships among dairy consumption, gut microbiota and cardiometabolic health. EBioMedicine 2021;66:103284. 33752125 10.1016/j.ebiom.2021.103284 PMC7985282

[CIT0100] Singh RK , Chang HW, Yan D et al. Influence of diet on the gut microbiome and implications for human health. J Transl Med 2017;15:1–17. 28388917 10.1186/s12967-017-1175-y PMC5385025

[CIT0101] Suez J , Cohen Y, Valdés-Mas R et al. Personalized microbiome-driven effects of non-nutritive sweeteners on human glucose tolerance. Cell 2022;185:3307–3328.e19. 35987213 10.1016/j.cell.2022.07.016

[CIT0102] Szabo de Edelenyi F , Philippe C, Druesne-Pecollo N et al. Depressive symptoms, fruit and vegetables consumption and urinary 3-indoxylsulfate concentration: a nested case-control study in the French Nutrinet-Sante cohort. Eur J Nutr 2021;60:1059–1069. 32588216 10.1007/s00394-020-02306-0

[CIT0103] Tigchelaar EF , Zhernakova A, Dekens JAM et al. Cohort profile: LifeLines DEEP, a prospective, general population cohort study in the northern Netherlands: study design and baseline characteristics. BMJ Open 2015;5:e006772. 10.1136/bmjopen-2014-006772 PMC4554905 26319774

[CIT0104] Trefflich I , Marschall H-U, Giuseppe R et al. Associations between dietary patterns and bile acids-results from a cross-sectional study in vegans and omnivores. Nutrients 2019;12:47. 31878000 10.3390/nu12010047 PMC7019893

[CIT0105] Tripathi A , Debelius J, Brenner DA et al. The gut–liver axis and the intersection with the microbiome. Nat Rev Gastroenterol Hepatol 2018;15:397–411. 29748586 10.1038/s41575-018-0011-z PMC6319369

[CIT0106] Valdes AM , Walter J, Segal E et al. Role of the gut microbiota in nutrition and health. BMJ2018a;361:k2179. 29899036 10.1136/bmj.k2179 PMC6000740

[CIT0107] Valdes AM , Walter J, Segal E et al. Role of the gut microbiota in nutrition and health. BMJ2018b;361:36–44. 10.1136/bmj.k2179 PMC6000740 29899036

[CIT0108] Van de Wouw M , Schellekens H, Dinan TG et al. Microbiota-gut-brain axis: modulator of host metabolism and appetite. J Nutr 2017;147:727–745. 28356427 10.3945/jn.116.240481

[CIT0109] Vanegas SM , Meydani M, Barnett JB et al. Substituting whole grains for refined grains in a 6-wk randomized trial has a modest effect on gut microbiota and immune and inflammatory markers of healthy adults. Am J Clin Nutr 2017;105:635–650. 28179226 10.3945/ajcn.116.146928 PMC5320415

[CIT0110] Vendrame S , Guglielmetti S, Riso P et al. Six-week consumption of a wild blueberry powder drink increases bifidobacteria in the human gut. J Agric Food Chem 2011;59:12815–12820. 22060186 10.1021/jf2028686

[CIT0111] Vetrani C , Costabile G, Luongo D et al. Effects of whole-grain cereal foods on plasma short chain fatty acid concentrations in individuals with the metabolic syndrome. Nutrition 2016;32:217–221. 26706023 10.1016/j.nut.2015.08.006

[CIT0112] Wan Y , Wang F, Yuan J et al. Effects of dietary fat on gut microbiota and faecal metabolites, and their relationship with cardiometabolic risk factors: a 6-month randomised controlled-feeding trial. Gut 2019;68:1417–1429. 30782617 10.1136/gutjnl-2018-317609

[CIT0113] Wang Z , Tang WH, Buffa JA et al. Prognostic value of choline and betaine depends on intestinal microbiota-generated metabolite trimethylamine-N-oxide. Eur Heart J 2014;35:904–910. 24497336 10.1093/eurheartj/ehu002 PMC3977137

[CIT0114] Wang DD , Nguyen LH, Li Y et al. The gut microbiome modulates the protective association between a Mediterranean diet and cardiometabolic disease risk. Nat Med2021a;27:333–343. 33574608 10.1038/s41591-020-01223-3 PMC8186452

[CIT0115] Wang D , Doestzada M, Chen L et al. Characterization of gut microbial structural variations as determinants of human bile acid metabolism. Cell Host Microbe2021b;29:1802–1814.e5. 34847370 10.1016/j.chom.2021.11.003

[CIT0116] Wang H , Gou W, Su C et al. Association of gut microbiota with glycaemic traits and incident type 2 diabetes, and modulation by habitual diet: a population-based longitudinal cohort study in Chinese adults. Diabetologia2022a;65:1145–1156. 35357559 10.1007/s00125-022-05687-5 PMC9174105

[CIT0117] Wang M , Wang Z, Lee Y et al. Dietary meat, Trimethylamine N-oxide-related metabolites, and incident cardiovascular disease among older adults: the Cardiovascular Health Study. Arterioscler Thromb Vasc Biol2022b;42:e273–e288. 35912635 10.1161/ATVBAHA.121.316533 PMC9420768

[CIT0118] Wang Y , Dong Q, Hu S et al. Decoding microbial genomes to understand their functional roles in human complex diseases. IMeta2022c;1:e14. 10.1002/imt2.14 PMC10989872 38868571

[CIT0119] Watson H , Mitra S, Croden FC et al. A randomised trial of the effect of omega-3 polyunsaturated fatty acid supplements on the human intestinal microbiota. Gut 2018;67:1974–1983. 28951525 10.1136/gutjnl-2017-314968

[CIT0120] Wedlake L , Slack N, Andreyev HJN et al. Fiber in the treatment and maintenance of inflammatory bowel disease: a systematic review of randomized controlled trials. Inflamm Bowel Dis 2014;20:576–586. 24445775 10.1097/01.MIB.0000437984.92565.31

[CIT0121] Wei H , Zhao M, Huang M et al. FMO3-TMAO axis modulates the clinical outcome in chronic heart-failure patients with reduced ejection fraction: evidence from an Asian population. Front Med 2022;16:295–305. 34159537 10.1007/s11684-021-0857-2

[CIT0122] Wu GD , Compher C, Chen EZ et al. Comparative metabolomics in vegans and omnivores reveal constraints on diet-dependent gut microbiota metabolite production. Gut 2016;65:63–72. 25431456 10.1136/gutjnl-2014-308209 PMC4583329

[CIT0123] Wu TT , Xiao J, Manning S et al. Multimodal data integration reveals mode of delivery and snack consumption outrank salivary microbiome in association with caries outcome in Thai children. Front Cell Infect Microbiol2022a;12:881899. 35677657 10.3389/fcimb.2022.881899 PMC9168266

[CIT0124] Wu Y , Gou W, Yan Y et al. Gut microbiota and acylcarnitine metabolites connect the beneficial association between equol and adiposity in adults: a prospective cohort study. Am J Clin Nutr2022b;116:1831–1841. 36095141 10.1093/ajcn/nqac252

[CIT0125] Xiao C , Wang JT, Su C et al. Associations of dietary diversity with the gut microbiome, fecal metabolites, and host metabolism: results from 2 prospective Chinese cohorts. Am J Clin Nutr 2022;116:1049–1058. 36100971 10.1093/ajcn/nqac178 PMC9535526

[CIT0126] Yu D , Shu XO, Rivera ES et al. Urinary levels of Trimethylamine-N-oxide and incident coronary heart disease: a prospective investigation among urban Chinese Adults. J Am Heart Assoc 2019;8:e010606. 30606084 10.1161/JAHA.118.010606 PMC6405718

[CIT0127] Yu D , Nguyen SM, Yang Y et al. Long-term diet quality is associated with gut microbiome diversity and composition among urban Chinese adults. Am J Clin Nutr 2021;113:684–694. 33471054 10.1093/ajcn/nqaa350 PMC7948864

[CIT0128] Zaramela LS , Martino C, Alisson-Silva F et al. Gut bacteria responding to dietary change encode sialidases that exhibit preference for red meat-associated carbohydrates. Nat Microbiol 2019;4:2082–2089. 31548686 10.1038/s41564-019-0564-9 PMC6879853

[CIT0129] Zeevi D , Korem T, Zmora N et al. Personalized nutrition by prediction of glycemic responses. Cell 2015;163:1079–1095. 26590418 10.1016/j.cell.2015.11.001

[CIT0130] Zheng J-S , Ordovás JM. Precision nutrition for gut microbiome and diabetes research: application of nutritional n-of-1 clinical trials. J Diabetes 2021;13:1059–1061. 34453774 10.1111/1753-0407.13220

[CIT0131] Zhernakova A , Kurilshikov A, Bonder MJ et al. Population-based metagenomics analysis reveals markers for gut microbiome composition and diversity. Science 2016;352:565–569. 27126040 10.1126/science.aad3369 PMC5240844

